# Challenges in Hip Replacement in Hip Dysplasia Cases and the "Happy Elephant Sign

**DOI:** 10.7759/cureus.2762

**Published:** 2018-06-07

**Authors:** MN Baig, Usman Baig, Bill Curtin

**Affiliations:** 1 Department of Trauma and Orthopaedic Surgery, Galway University Hospital, Galway, IRL; 2 Medicine, Bahawal Victoria Hospital, Bahawalpur., Bahawalpur, PAK

**Keywords:** hip dysplasia, modular hip

## Abstract

Hip dysplasia is an abnormal development of the hip that consists of a spectrum of different abnormalities featuring an abnormal relation of the femoral head to the acetabulum. It can be treated in early childhood when it is diagnosed. Later in adult life, it is more challenging. We present a case of a 50-year-old woman who presented to us with adult hip dysplastic changes; we undertook the care of her left hip and treated her surgically.

## Introduction

Developmental dislocation (dysplasia) of the hip (DDH) is a spectrum consisting of acetabulum dysplasia, femoral head subluxation, and femoral head dislocation. The etiology is multifactorial, including ligament laxity, intrauterine factors, and genetic predisposition [1]. The classifications for hip dysplasia in adults commonly used are either Hartofilakidis classification or the Crowe classification [2].

Due to their altered hip anatomy, adults who have DDH tend to develop arthritis of the hip at an early age as compared to the general population.

## Case presentation

We describe the case of a 50-year-old woman who presented with adult hip dysplasia of high dislocation (Hartofilakidis Classification, type C) /Crowe grade IV. She is a known smoker and has hypertension. She had been tolerating mild pain for the past few years, but recently it had become debilitating. Additionally, there was a leg length discrepancy making the affected left side 7 cm shorter.

When she initially presented a few years ago, she was started on physical therapy and analgesia as she did not want to consider surgical intervention. However, the pain had become unbearable, and the functionality of the hip was compromised.

**Figure 1:**
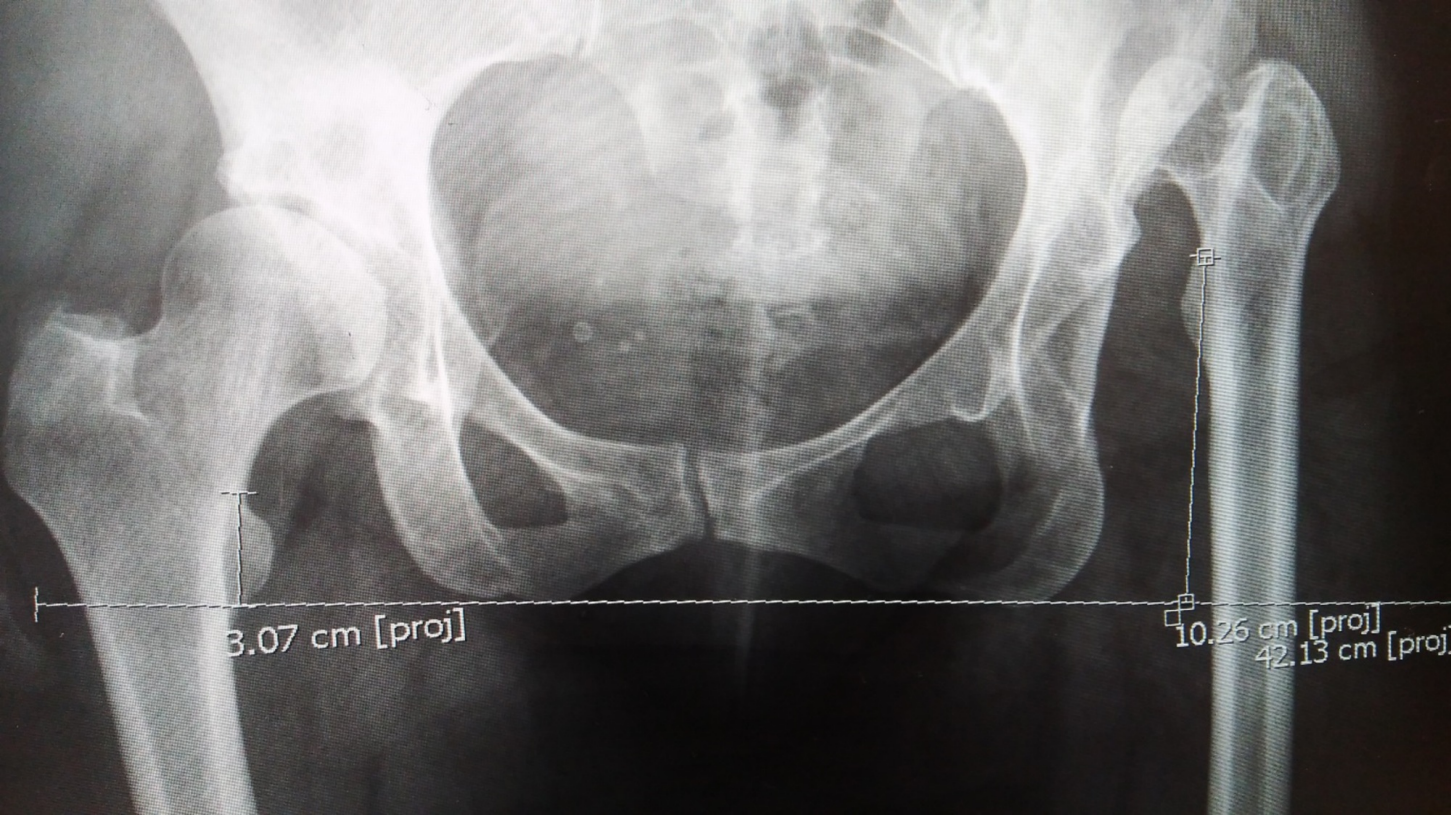
X-ray of the pelvis Pre-operative X-ray showing hip dysplasia and leg shortening.

**Figure 2:**
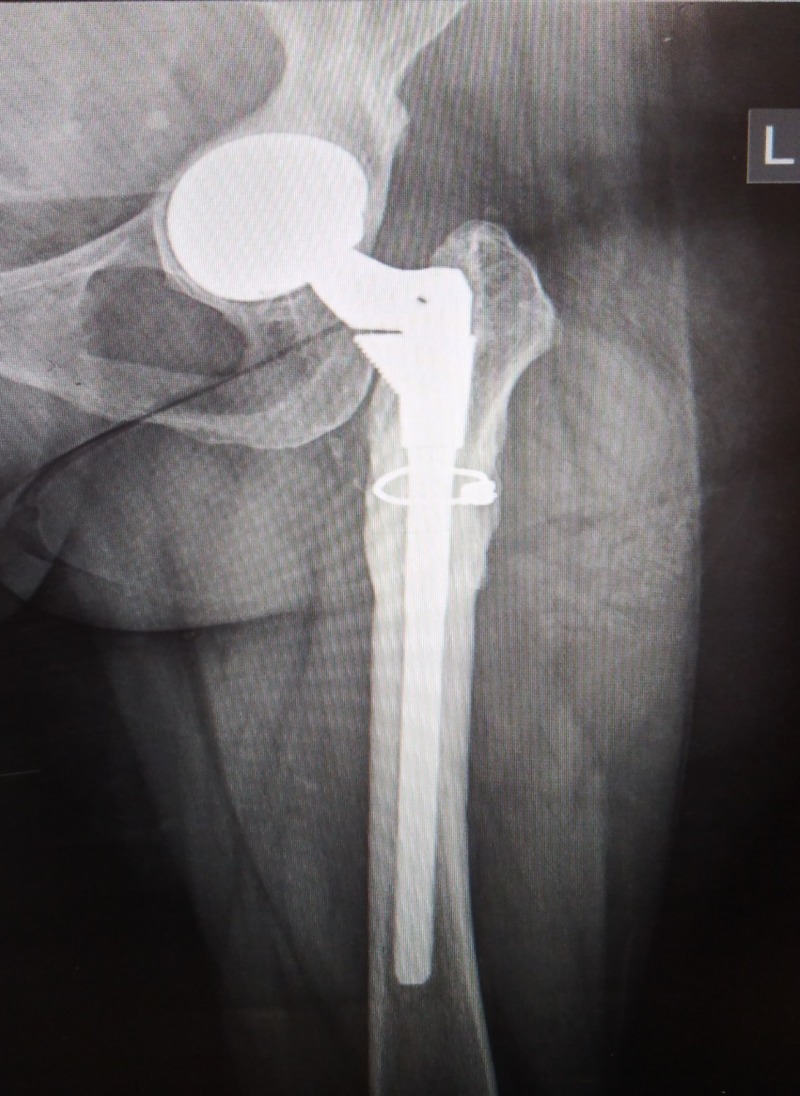
Post-operative X-ray Post-operative modular hip replacement.

We recommended a hip replacement and discussed the potential benefits and risk factors. It was explained to the patient that in dealing with the developmental dislocation we would have to recreate normal hip mechanics which requires positioning of the acetabular component in hemispherical acetabular cavity at the centre of rotation. It would also entail placing a femoral component at a much lower level within a femoral canal which was abnormally narrow. She underwent the left hip replacement which was surgically challenging. A special hip implant was used for the hip replacement. It was a modular implant typically used for difficult cases where there is anatomical distortion. The surgery was completed without any complications. We were able to correct the leg length discrepency by 4cm. Following surgery the patient underwent physical therapy and is now independently mobile without the use of external support and has no functional difficulties in day to day activities, (Figures 1-2).

## Discussion

Developmental hip dysplasia is the most common cause of secondary osteoarthritis of the hip especially in the younger age group [3]. There are anatomical abnormalities both in the acetabulum and femur depending on the severity of the dysplasia [4-5]. The common challenges encountered in dysplastic hip replacement include [6]:

-          Previous acetabular osteotomy

-          Previous femoral osteotomy

-          Abnormal femoral neck anteversion

-          Narrow femoral canal with reduced dimensions in the mediolateral and anteroposterior planes

-          Leg length discrepancy

There are three important points to consider in this case. First, the adult hip dysplasia; its presentation was challenging, and its complex severity caused arthritis in this relatively younger patient. Secondly, there are important considerations when offering these patients surgical treatment options as the hip replacement is difficult for both the patient and the surgeon. The surgery demands a large amount of planning, anticipation of complications, and overall maximum effort to restore the biomechanics of the replacement hip joint to be as close to the normal hip as possible. Finally, considering the modular hip in general and, more specifically, in patients with hip dysplasia. The modular hip replacement is a relatively inexpensive implant that allows for the option of mixing and matching the stem, neck, and offset according to the individual anatomic demands of the patient.

The senior author is an experienced surgeon who has been using the modular hip implants successfully in dysplastic hip replacements. The modularity helps in achieving and correcting the different abnormal parameters encountered in dysplastic hip including femoral neck anteversion, offset, and leg length. It also provides desired proximal and distal implant stability as compared to conventional implants [6].

**Figure 3:**
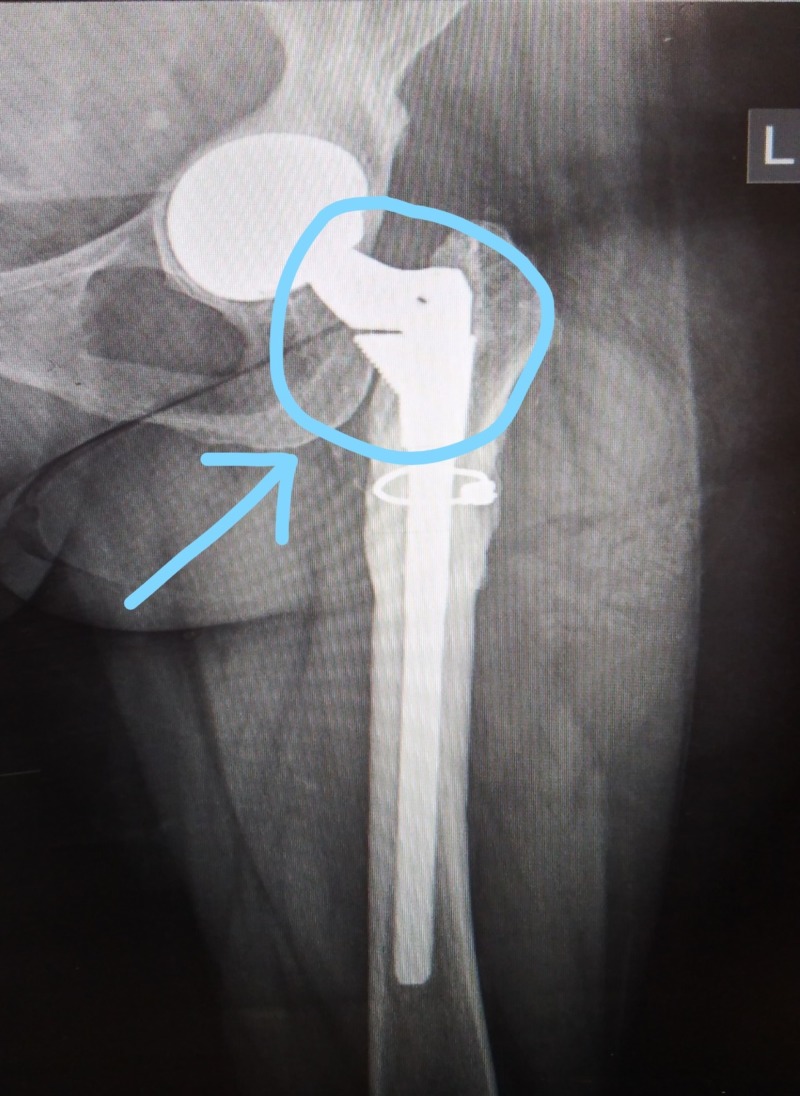
Post-operative X-ray with the 'happy elephant sign'

On a lighter note, the implant which is shown in the above-mentioned patient is a modular implant (S-ROM modular hip system) system, if you look at it closely, it looks like a happy elephant. So we call it a “Happy Elephant sign” (Figure 3).

## Conclusions

DDH is a complex abnormality. Correction requires a specific set of skills, special implants, and an enthusiastic patient who will fully cooperate with essential postoperative rehabilitation. Modular implant is a good choice of implant for complex DDH hip replacement surgery as it helps to attain desirable results in an abnormal hip anatomy.
